# Germline Variants Associated with Nasopharyngeal Carcinoma Predisposition Identified through Whole-Exome Sequencing

**DOI:** 10.3390/cancers14153680

**Published:** 2022-07-28

**Authors:** Ning-Yuan Lee, Melissa Hum, Pei-Yi Ong, Matthew Khine Myint, Enya H. W. Ong, Kar-Perng Low, Zheng Li, Boon-Cher Goh, Joshua K. Tay, Kwok-Seng Loh, Melvin L. K. Chua, Soo-Chin Lee, Chiea-Chuen Khor, Ann S. G. Lee

**Affiliations:** 1 Division of Cellular and Molecular Research, Humphrey Oei Institute of Cancer Research, National Cancer Centre Singapore, Singapore 196910, Singapore; lee.ning.yuan@nccs.com.sg (N.-Y.L.); melissa.hum.w.c@nccs.com.sg (M.H.); nccmkm2019@gmail.com (M.K.M.); 2 Department of Hematology-Oncology, National University Cancer Institute, Singapore (NCIS), National University Health System, Singapore 119074, Singapore; pei_yi_ong@nuhs.edu.sg (P.-Y.O.); boon_cher_goh@nuhs.edu.sg (B.-C.G.); csilsc@nus.edu.sg (S.-C.L.); 3 Division of Medical Sciences, National Cancer Centre Singapore, Singapore 196910, Singapore; enya.ong.h.w@nccs.com.sg (E.H.W.O.); lowkarperng@gmail.com (K.-P.L.); gmsclkm@nus.edu.sg (M.L.K.C.); 4 Genome Institute of Singapore, A-STAR, Singapore 138672, Singapore; liz11@gis.a-star.edu.sg (Z.L.); khorcc@gis.a-star.edu.sg (C.-C.K.); 5 Department of Medicine, Yong Loo Lin School of Medicine, National University of Singapore, Singapore 117597, Singapore; 6 Cancer Science Institute, Singapore (CSI), National University of Singapore, Singapore 117599, Singapore; 7 Department of Otolaryngology—Head & Neck Surgery, National University Hospital, Singapore 119074, Singapore; joshtay@nus.edu.sg (J.K.T.); entlks@nus.edu.sg (K.-S.L.); 8 Department of Otolaryngology—Head & Neck Surgery, National University of Singapore, Singapore 119228, Singapore; 9 Department of Head and Neck and Thoracic Cancers, Division of Radiation Oncology, National Cancer Centre Singapore, Singapore 196910, Singapore; 10 SingHealth Duke-NUS Oncology Academic Clinical Programme (ONCO ACP), Duke-NUS Graduate Medical School, Singapore 117593, Singapore; 11 Department of Physiology, Yong Loo Lin School of Medicine, National University of Singapore, Singapore 117593, Singapore

**Keywords:** nasopharyngeal carcinoma, exome sequencing, germline variants, genetic predisposition

## Abstract

**Simple Summary:**

The aim of this study was to identify the germline genetic variants associated with an increased risk of developing nasopharyngeal carcinoma (NPC). DNA samples from 119 Singaporean NPC patients were sequenced, with 17 pathogenic variants in 17 genes found to be enriched in NPC patients as compared to unaffected controls. Five of these variants (in the *JAK2, PRDM16*, *LRP1B*, *NIN*, and *NKX2-1* genes) were supported by repeated testing on an independent set of Singaporean NPC patients and unaffected Singaporean controls. A *FANCE* variant was observed in two siblings with NPC, but not in three unaffected siblings of the same family. Gene-based burden testing recapitulated the association between *NKX2-1* and *FANCE* variants with NPC risk. Pathway analysis revealed a higher frequency of germline mutations in endocytosis and immune-modulating pathways. Our research has identified new variants and genes associated with susceptibility to NPC, which are relevant for an improved understanding of the genetic predisposition of NPC.

**Abstract:**

The current understanding of genetic susceptibility factors for nasopharyngeal carcinoma (NPC) is still incomplete. To identify novel germline variants associated with NPC predisposition, we analysed whole-exome sequencing data from 119 NPC patients from Singapore with a family history of NPC and/or with early-onset NPC, together with 1337 Singaporean participants without NPC. Variants were prioritised and filtered by selecting variants with minor allele frequencies of <1% in both local control (n = 1337) and gnomAD non-cancer (EAS) (n = 9626) cohorts and a high pathogenicity prediction (CADD score > 20). Using single-variant testing, we identified 17 rare pathogenic variants in 17 genes that were associated with NPC. Consistent evidence of enrichment in NPC patients was observed for five of these variants (in *JAK2, PRDM16, LRP1B, NIN*, and *NKX2-1*) from an independent case-control comparison of 156 NPC patients and 9770 unaffected individuals. In a family with five siblings, a *FANCE* variant (p. P445S) was detected in two affected members, but not in three unaffected members. Gene-based burden testing recapitulated variants in *NKX2-1* and *FANCE* as being associated with NPC risk. Using pathway analysis, endocytosis and immune-modulating pathways were found to be enriched for mutation burden. This study has identified NPC-predisposing variants and genes which could shed new insights into the genetic predisposition of NPC.

## 1. Introduction

Nasopharyngeal carcinoma (NPC) afflicted 129,000 new patients globally in 2018 [1,2]. However, the global incidence of NPC is not homogenous, with more than two thirds of new NPC cases occurring in East Asia and Southeast Asia. Epstein–Barr virus (EBV) infection is the most common causative factor of NPC, although other environmental risk factors, such as the consumption of preserved foods, alcohol, and poor oral hygiene, have also been associated with NPC risk [3]. Recently, a meta-analysis of 334,935 men demonstrated a dose–response relationship between smoking and NPC risk, providing support for smoking as yet another risk factor for NPC [4,5]. Prospective studies in Singapore and China have shown a from two- to more than a ten-fold increased risk of NPC in first degree relatives of patients [6,7,8,9], suggesting a genetic component in the development of NPC.

Potential genetic factors for NPC based on various models of tumorigenesis have implicated developmental genes such as *CRIP2* and *MIPOL1*, EBV oncoproteins *LMP1* and *LMP2*, and increased EBV-associated tumorigenesis via specific EBV-infection-prone HLA haplotypes [10]. More recent studies have employed next-generation sequencing (NGS) to examine the genetics of NPC and identify germline variants in NPC. Using whole-exome sequencing (WES) of NPC tumour DNA samples, somatic variants have been identified in the Ras and cell cycle pathways, *NF-kB* pathway and *MLL3* gene [11,12,13,14]. WES of germline DNA from NPC patients of southern Chinese descent has also identified germline variants associating *MST1R* and *RPA1* with a genetic predisposition for NPC and aggressive disease, respectively [15,16]. In addition, germline mutations associated with increased cancer risk have been reported in over 100 genes [17], and targeted sequencing has found variants in suspected familial or sporadic NPC susceptibility genes *CDKN2A/2B*, *BRD2*, *TNRFRSF19*, and *CLPTM1L/TERT* [18].

Here, we performed WES on germline DNA from 119 NPC patients from Singapore, to determine the prevalence of mutations in previously reported NPC-associated genes and identify new NPC susceptibility variants. The variants identified in this cohort were also examined in an independent cohort of young 156 NPC cases who were diagnosed before they reached 40 years old. In addition, case-control association analyses were performed against local control and gnomAD non-cancer East Asian control cohorts. By using a stringent filtering and prioritisation strategy, we identified germline variants in *FANCE*, *JAK2, PRDM16, LRP1B, NIN,* and *NKX2-1* that may be implicated in the pathogenesis of NPC. Pathway analysis revealed that the endocytosis and immune-modulating pathways were enriched for mutation burden.

## 2. Materials and Methods

### 2.1. Study Participants

**Figure 1:**
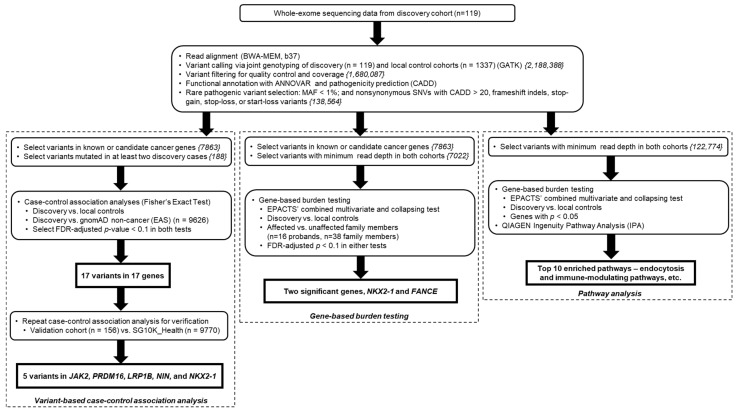
Study design and steps taken for the selection of candidate NPC susceptibility variants. For each variant filtering step, the number of variants remaining after filtering is given within the curly brackets.

Blood samples for the discovery cohort were obtained from 119 patients who were diagnosed with NPC from the National University Hospital, Singapore (Figure 1). These patients either had early-onset NPC (at or below 40 years of age) and/or a family history of NPC in first- and/or second-degree relatives (Supplementary Table S1). Blood samples were also obtained from 38 family members (2 affected, 36 unaffected) of 16 probands from the discovery cohort. Unaffected control individuals comprised 1337 Singaporean participants who also underwent whole-exome sequencing.

For the validation cohort, blood samples were obtained from 156 patients diagnosed with NPC at or below 40 years of age from the National Cancer Centre Singapore (Supplementary Table S1). Unaffected control individuals comprised 9770 healthy Singaporean participants (SG10K_Health) [19]. All study participants provided written informed consent and the study was approved by the Institutional Review Boards at the study sites.

### 2.2. Whole-Exome Sequencing

For the discovery cohort and their family members, genomic DNA was extracted from peripheral blood mononuclear cells using routine laboratory methods [20]. Sequencing libraries were prepared from the DNA samples using the Agilent SureSelect Human All Exon V6 kit (Agilent Technologies, Santa Clara, CA, USA) and were 150bp paired-end sequenced on the Illumina NovaSeq 6000 platform (Illumina, San Diego, CA, USA). For the validation cohort, sequencing libraries were prepared using the Agilent SureSelect Human All Exon V6 +UTR kit (Agilent Technologies, Santa Clara, CA, USA). Control cohorts were prepared using similar, well-described protocols and sequenced using 150bp paired-end end reads on Illumina high-throughput instruments at 100X (NovaSeq 6000 or HiSeq 4000) (Illumina, San Diego, CA, USA).

### 2.3. Germline Variant Discovery and Annotation

For each sequenced sample, read pairs were aligned to the human reference genome (b37) using BWA-MEM (v0.7.17) [21]. The reads were sorted and reads from multiple lanes were merged with SAMtools (v1.9) [22]. PCR duplicates were flagged for filtering downstream using MarkDuplicates in GATK v4.1.9.0 [23]. Base quality recalibration was carried out and applied using GATK’s BaseRecalibrator and ApplyBQSR. Subsequently, variant calling was performed with the GATK HaplotypeCaller producing a GVCF file for each sample. Joint genotyping was performed alongside GVCFs of the local control cohort with the GenotypeGVCFs function. Low-quality variants were removed, using the recommended hard filters from gnomAD v2.1 [24].

Variants were annotated with ANNOVAR [25], including in silico prediction tools from CADD [26], SIFT [27], PolyPhen-2 [28], and MutationTaster [29], as well as the ClinVar database [30]. ACMG-AMP pathogenicity annotations were obtained via InterVar [31,32] (Figure 1).

### 2.4. Prioritisation and Filtering of Variants

Potential NPC variants mutated in two or more affected patients were first selected from the jointly genotyped variants. Rare variants were selected by filtering for variants with minor allele frequency (MAF) less than 1% in both the gnomAD non-cancer (EAS) and the local control cohorts. Then, pathogenic variants were selected from nonsynonymous variants with CADD v1.3 phred score greater than 20. This stringent CADD threshold represents the top 1% of CADD-predicted pathogenic variants. Loss-of-function variants (frameshift insertions and deletions, stop-gains, stop-losses, or start-losses) were retained. Finally, rare pathogenic variants in known cancer genes were selected by choosing variants in genes appearing in at least two of the following cancer gene databases or literature sources: Network of Cancer Genes (NCG) 6.0 [33], COSMIC Cancer Gene Census v94 (CGC) [34], germline cancer predisposition genes [17], cancer driver genes [35], or cancer driver genes inferred from nucleotide context [36].

### 2.5. Case-Control Association Analysis

Principal component analysis (PCA) was performed to verify that participants with NPC and unaffected local control participants are of a similar genetic ancestry (Supplementary Figure S1). PCA was done using SNPRelate using default parameters [37]. Case-control association analysis was performed with variants in known cancer genes by comparing their allele frequency in the discovery cohort with that in the local control cohort and gnomAD non-cancer (EAS) (n = 9626). Variants not reported in gnomAD were assumed to have zero allele count with a linearly interpolated allele number, if they were within 300 nucleotides of a valid gnomAD variant. Variants which were significantly more common (FDR-adjusted *p*-value less than 0.10) in both comparisons were selected.

Case-control association analysis was repeated using a validation cohort of 156 NPC patients from Singapore, and the SG10K_Health control cohort (release 5.3) comprising of 9770 Chinese, Malay, and Indian healthy volunteers from Singapore [19].

### 2.6. Segregation Analysis

Germline variants from family members of discovery cohort probands were called using DRAGEN v3.8.4 with hg38 as reference genome [38]. Variants were filtered for quality control using default DRAGEN filters, then lifted over to hg19. Variant annotation and filtering for pathogenicity was performed using the same methods as described for the discovery and local control cohorts.

### 2.7. Gene-Based Burden Testing

Gene-based burden testing was used to compare the proportion of affected patients with rare pathogenic variants in known cancer genes in the discovery cohort versus local control cohort. Differences in sequencing coverage were controlled for by setting an average per-cohort minimum read depth cut-off. A minimum average cut-off of 25.1 reads per sample in the local control cohort was chosen to balance between test validity, as quantified by QQ-plot *R*^2^, and the number of variants to be filtered (Supplementary Figure S2A). PCA covariates were not included in the test as they did not appear to improve the validity of the test (Supplementary Figure S2B). The test was performed using the combined multivariate and collapsing test implemented by EPACTS’ emmaxCMC [39].

To verify variants identified in 17 genes prioritised from variant-based analysis, the gene-based burden test was also performed on 16 probands and their 38 family members for all pathogenic variants.

### 2.8. Pathway Analysis

The gene-based burden test on discovery and local controls was repeated with the same parameters, without filtering for known cancer genes. Then, 559 genes with *p* < 0.05 were analysed using QIAGEN Ingenuity Pathway Analysis (IPA) (QIAGEN, Redwood City, CA, USA) for enriched pathways. Enrichment *p*-values for canonical pathways were calculated using the right-tailed Fisher’s Exact Test.

### 2.9. Variant Quality Checks with Integrative Genomics Viewer (IGV)

Low-quality variants in both variant- and gene-based results were identified by checking their alignments in IGV [40] (Supplementary Figure S3). For the variant-based results, the problematic variants were removed from the list of results. For the gene-based results, the gene-based tests were re-run with the exclusion of the problematic variants.

### 2.10. Statistical Analysis

Variant-based case-control analyses were performed using a two-tailed Fisher’s Exact Test [41]. Gene-based burden tests were performed via the combined multivariate and collapsing test using EPACTS emmaxCMC [39]. *p*-values were corrected for multiple testing to reduce using the Benjamini–Hochberg method to reduce the false discovery rate [42].

## 3. Results

### 3.1. Characteristics of Study Participants

In the discovery cohort, patients either had an age at diagnosis of 40 years of age or younger (53/119 or 58.9%), first- or second-degree family history of any cancer (29/119 or 24.4%), or both (37/119 or 31.1%). In the validation cohort, all patients had an age at diagnosis of 40 years of age or younger. Both cohorts were predominantly ethnic Chinese: the discovery cohort comprised 105 ethnic Chinese (88.2%), 3 ethnic Malays (2.5%), 1 ethnic Indian (0.8%), and 10 NPC patients (8.4%) of other ethnicities; the validation cohort had 132 ethnic Chinese (84.6%), 9 ethnic Malays (5.8%), and 15 patients (9.6%) of other ethnicities (Supplementary Table S1).

### 3.2. Variant Filtering

The 119 discovery cohort patients and 1337 local controls were jointly genotyped using GATK (Figure 1). In total, 1,680,087 variants in both the discovery cohort or local controls passed the filtering criteria of excess heterozygosity (as in the expected Hardy–Weinberg equilibrium), read depth, allele balance and genotype quality. Of these, 272,536 variants were recurrent, being present in two or more cases. After filtering for variants with minor allele frequency (MAF) less than 1% in both gnomAD (EAS) and local control cohorts with CADD v1.3 PHRED score larger than 20, frameshift insertions and deletions, stop-gains, stop-losses, or start-losses, a final list of 188 rare pathogenic variants belonging to genes in known cancer genes according to cancer gene databases (COSMIC, NCG) [33,34] or literature sources [17,35,36] were selected for further case-control association analysis.

A list of singleton variants, each present only in one case, are shown in Supplementary Table S2. These variants were excluded from the variant-based tests, but not the gene-based tests.

### 3.3. Variant-Based Case-Control Association Analysis

We performed case-control association analysis on 188 rare pathogenic variants in known cancer genes, comparing their allele frequencies in our discovery case cohort versus both local controls and gnomAD (EAS). Of these 188 variants, we shortlisted 17 variants, all of which were non-synonymous SNVs with a CADD PHRED score greater than 20, in 17 cancer-associated genes with substantially higher allele frequencies in patients with NPC as compared to both local control and gnomAD cohorts (Table 1, Supplementary Table S3). These include variants in genes encoding FANCE, a subunit of the Fanconi Anaemia (FA) nuclear complex; NKX2-1, a transcription factor and negative regulator of the NF-κB signalling pathway [43]; and other recognized oncogenes *JAK2, PRDM16, BMPR1A* and tumor-suppressor genes *KMT2C, FAT4, and LRP1B* [34] (Supplementary Tables S3 and S4). A plot showing the frequency of these 17 variants, together with the age group and family history for each case, is shown in Supplementary Figure S4.

An independent case-control association was repeated for these 17 variants, using a validation cohort of early-onset NPC patients (n = 156) and healthy controls from SG10K_Health (n = 9770). Five of the 17 variants, in *JAK2, PRDM16, LRP1B, NIN,* and *NKX2-1*, were also present in the validation cohort. All five variants were more common in patients with NPC compared to the SG10K_Health controls (Table 2).

### 3.4. Gene-Based Burden Testing Shows Increased Mutation Burden for NKX2-1 and FANCE

To determine if the same case-control associations are reflected at the gene-level, we performed gene-based burden testing on rare pathogenic variants in known cancer genes, comparing the germline mutation burden in discovery cases versus local controls (Figure 1). Gene-based burden testing results showed an association between *NKX2-1* and NPC risk, as two of 119 cases (1.7%) but none of the 1337 controls had variants in *NKX2-1* (FDR-adjusted *p* = 0.0144) (Table 3).

For 16 probands from the discovery cohort, DNA samples were available from two NPC-affected family members, and 36 unaffected family members. The gene-based burden test was used to verify variants from the variant-based association test results. In this test, *FANCE* had a significantly larger germline mutation burden, where two affected individuals (11.1%) but no unaffected individuals had a *FANCE* variant (rs141551053) (Table 4). The two affected individuals with this *FANCE* variant are siblings: the proband A0118 and affected brother A0118-4. A0118 has three other siblings, all of whom are unaffected by NPC and did not carry this *FANCE* variant.

### 3.5. Differential Gene Expression in Primary Tumor Versus Normal Tissue

We further checked the gene expression in primary tumor versus normal tissue for genes *JAK2, PRDM16, LRP1B, NIN,* and *NKX2-1* with variants repeatedly enriched in variant-based analysis, and, *FANCE*, enriched in gene-based burden testing, using the TCGA database (Supplementary Figure S5). Except for *JAK2* and *NKX2-1*, all genes were differentially expressed in primary head and neck tumors as compared to normal tissue. All six genes were differentially expressed in primary tumor versus normal tissue expression, in at least one of five commonly diagnosed cancers [1].

### 3.6. Pathway Analysis Suggests the Involvement of the Endocytosis and Immune-Modulating Pathways

We performed pathway analysis using significant genes in a gene-based burden test of variants between discovery case and local control cohorts. The top ten canonical pathways based on the significance of enrichment *p*-values are shown in Table 5 and Supplementary Figure S6. Two possible mechanisms for EBV entry into the cell, the clathrin- and caveolar-mediated endocytosis signalling pathways [44,45], were enriched for mutation burden (*p* = 0.0274 and *p* = 0.0441 respectively). Immune-modulating pathways were also significantly enriched, particularly GM-CSF signalling, “JAK1 and JAK3 in γc cytokine signalling”, and IL-15 production pathways (*p* = 0.0092, *p* = 0.0291, and *p* = 0.0357, respectively).

Odds ratios and *p*-values were also calculated for NPC patients and controls with variants in any genes in each of the implicated pathways. Our results show that all ten pathways were significantly enriched in our dataset (OR = 3.1–70.4, *p* < 0.05) (Table 5).

### 3.7. Variants and Genes Implicated in Prior Literature

We were able to replicate the results of six variants and four genes previously implicated in NPC by previous studies [11,12,13,15,16,18,46,47,48,49,50,51]. Four common SNVs in *GABBR1*, encoding a subunit of the GABA receptor, which were previously associated with NPC, were replicated in our cohort at *p* = 0.05 [46,47]. We also replicated two variants in the transcription regulator gene *BRD2* [18,48], though this variant was not included in our primary variant-based case-control association test as *BRD2* did not satisfy the criterion of being a known cancer gene in cancer gene databases or the literature sources (Supplementary Table S5). Four genes previously associated with NPC, *BRD2, CTNNB1, TRMT10B,* and *IRF5,* were also replicated in our cohort’s gene-based burden test (*p* < 0.0394) [11,15,18] (Supplementary Table S6).

## 4. Discussion

To identify novel germline variants predisposing one to NPC, we first examined WES of 119 Singaporean patients who had early-onset NPC and/or a family history of NPC, followed by an independent set of 156 early-onset NPC patients. Here, we discovered an initial list of 17 unique variants in 17 genes associated with NPC, five of which (in *JAK2, PRDM16, LRP1B, NIN*, and *NKX2-1*) were also associated with NPC in the validation case cohort.

Of these 17 variants, two variants were previously reported in cancer-related studies. The *BMPR1A* nonsynonymous SNV (rs55932635) was identified in one of 56 *BRCA*-negative breast cancer patients in Puerto Rico [52], while the *JAK2* nonsynonymous SNV (rs200018153) was found in one of 1487 acute myeloid leukaemia (AML) patients in the United States [53]. The remaining 15 variants, to the best of our knowledge, have not been implicated with cancer, based on a literature search of their RefSNP numbers (Table 3).

We observed that some variants were absent from control cohorts. For example, the *APOB* variant was absent from all three control cohorts (Table 1 and Table 2). Additionally, the *FAT3* and *ZEB1* variants were absent from two of three control cohorts. In addition, 12 of the 17 variants have not been reported in ClinVar. This could be due to the underrepresentation of Asian variants in the ClinVar database and underscores the necessity for more extensive sequencing of genomes from Asian populations.

Notably, in a family with five siblings, the *FANCE* nonsynonymous SNV (rs141551053) was detected in two affected siblings but not in three unaffected siblings. Furthermore, gene-based burden testing on 16 probands and 38 family members also showed an association between the *FANCE* gene and NPC. *FANCE* encodes a critical subunit of the Fanconi Anaemia (FA) nuclear complex [54], which facilitates DNA repair, replication, and chromosome segregation. The rs141551053 SNV alters an amino acid (P445S) in its C-terminal domain. Heterozygous mutations in FA genes have been associated with various cancer predispositions, including breast, ovarian, brain, and soft tissue cancers [54]. While heterozygous FA gene mutations have not been directly linked to NPC, patients with the autosomal recessive FA syndrome have a much higher risk of developing head and neck squamous cell carcinomas [55].

*NKX2-1* was identified from both variant-based case-control association analysis and gene-based burden testing of the discovery cohort, suggesting that it is associated with NPC predisposition. NKX2-1 is a homeobox transcription factor expressed in the adult thyroid, lung, bronchus, and nasopharynx [56]. In lung adenocarcinoma, NKX2-1 has a dual context-dependent tumour-suppressive or -promoting role [57]. Although NKX2-1 has not been linked to NPC predisposition, its genomic loci on chromosome arm 14q form a commonly deleted locus in NPC [51,58,59,60,61]. Furthermore, NKX2-1 has been observed to downregulate IKKβ in lung adenocarcinoma [43]. IKKβ is an activator of the NF-κB signaling pathway, and the NF-κB signaling pathway is a commonly activated pathway in NPC [62].

Finally, we identified a *JAK2* nonsynonymous SNV c.1174G>A (rs200018153) that was enriched in our discovery cohort. This variant was also detected in an independent validation cohort but did not reach statistical significance. Nonetheless, this *JAK2* variant had the highest allele frequency of all our 17 variants, in both discovery and validation cohorts. JAK2 is a non-receptor tyrosine kinase, and plays an important role in regulating the JAK/STAT signalling pathway that controls cell proliferation, differentiation, survival, and cytokine-mediated immune responses [63]. Mutations in *JAK2*, which lead to the hyperactivation of the JAK/STAT pathway, have been observed in many cancer types [64,65,66]. The rs200018153 SNV alters an amino acid (V392M) in the Src homology 2 (SH2) domain of JAK2. The most frequent and well-studied *JAK2* mutation is *JAK2-V617F*, which has been reported to be associated with predisposition to myeloproliferative disorders [67,68]. Other *JAK2* somatic mutations found in exon 12, R683 and T875, have also been linked to hematological malignancies [69,70,71]. Although little has been reported on *JAK2* mutations in NPC, two research groups have identified amplifications in the *JAK2* gene that are responsible for promoting cell proliferation and cell signalling in NPC [50,72]. Moreover, JAK2 has been found to be overexpressed in NPC tissues and high JAK2 expression correlates with poor clinical outcome [73].

There is conflicting evidence on whether the cellular entry of EBV is facilitated by clathrin-mediated or caveolar-mediated endocytosis, or both [44,45]. In our pathway analysis, both clathrin- and caveolar-mediated endocytosis signalling pathways were enriched for germline mutation burden in NPC. We also found enrichment in important immune-modulating pathways: the GM-CSF signalling pathway, which has been implicated in the recruitment of tumor-associated macrophages in NPC [74]; IL-15 production and its downstream JAK1/JAK3-related γc cytokine signalling pathways, which modulate both antiviral and antitumor effects [75] (Table 5).

In recent years, various genomic approaches have been used to interrogate the genetic landscape of NPC susceptibility. For example, SNP genotyping studies have reported germline polymorphisms in the MHC class I and nearby genes such as *GABBR1* and *HLA-F,* where such polymorphisms correlated with NPC risk [46,47]. A more recent exome-wide association study of 31,870 common SNPs involving 5553 patients with NPC has also identified a novel germline polymorphism in *RPA1* (rs1131636) conferring tumor progression and therapeutic resistance in NPC, which ultimately affects the patient’s survival [16]. *BRCA2* germline alterations related to homologous recombination deficiency were found to be associated with poor clinical outcome in patients with NPC [76]. Recently, WES has been increasingly applied to the discovery of cancer predisposition genes associated with NPC. A Japanese study has identified familial NPC-predisposing germline mutations in *MLL3* (*KMT2C*) in three family members of Italian descent [14]. Another WES analysis of germline DNA has also found NPC-associated rare variants in several genes, including *BRD2*, a chromatin remodelling gene, in Taiwanese NPC families and sporadic NPC cases [48]. Furthermore, two WES studies have revealed germline variants suggesting *MST1R* as a candidate susceptibility gene for NPC [15,77]. Along with *MST1R*, Dai and colleagues from Hong Kong identified several candidate NPC-susceptibility genes, including *TRMT10B*. In this study, we evaluated the prevalence of these variants and genes in our NPC discovery cohort, replicating the association between NPC and six variants in *BRD2* and *GABBR1*, and four genes (*BRD2, CTNNB1, TRMT10B,* and *IRF5*).

As a study of rare variants, our analysis is limited by its sample size. This study had a discovery cohort of 119 NPC cases, and a validation cohort of 156 cases. Further, larger association studies will be necessary to detect rarer variants with greater certainty. For example, some of the variants and genes from previous NPC studies were present in our cohorts but failed to reach statistical significance (*p* < 0.05), perhaps due to an insufficient sample size. Additional studies in larger cohorts of different genetic ancestry and meta-analyses are required to assess the frequency and importance of these NPC susceptibility variants.

## 5. Conclusions

In summary, we identified 17 germline variants in 17 genes that are associated with NPC predisposition. Six of these variants in six genes *JAK2, PRDM16, LRP1B, NIN, NKX2-1* and *FANCE* were further supported by at least one of four additional tests: an additional variant-based case-control association analysis on an independent validation cohort, gene-based testing on the original discovery cohort; gene-based testing and co-segregation analysis of family members of the discovery cohort. Our study provides new insights into the genetic susceptibility to NPC, which will facilitate further investigations with the potential to translate the findings into future clinical practice. This warrants further functional characterization of the *FANCE, NKX2-1,* and *JAK2* variants and elucidation of the mechanisms for the role of these genes in NPC development.

## Figures and Tables

**Table 1:** Allele frequencies and case-control association analysis of 17 variants in 17 known or candidate cancer genes identified from the discovery cohort.

Gene	Variant	Allele Frequency in Discovery Cohort (n = 119)	Minor Allele Frequency		Case-Control against Local Control Cohort			Case-Control against gnomAD (EAS)		
			Local Control Cohort (n = 1337)	gnomAD (EAS) (n = 9626)	Odds Ratio (5–95% CI)	*p*-Value	FDR Adjusted *p*-Value	Odds Ratio (5–95% CI)	*p*-Value	FDR Adjusted *p*-Value
*JAK2*	NM_004972.3:c.1174G>A	3.782%	0.748%	0.789%	5.2 (2.1–12.1)	0.000327	0.0268	4.9 (2.2–9.8)	0.000165	0.00387
*APOB*	NM_000384.3:c.6698T>C	0.840%	0.000%	0.000%	Inf (2.1–Inf)	0.00665	0.0606	Inf (15.2–Inf)	0.000149	0.00387
*FAT3*	NM_001008781.2:c.8983C>A	0.840%	0.000%	0.000%	Inf (2.1–Inf)	0.00665	0.0606	Inf (14.9–Inf)	0.000155	0.00387
*PRDM16*	NM_022114.4:c.2062G>A	1.261%	0.037%	0.042%	34.0 (2.7–1770.4)	0.00203	0.0606	30.0 (5.1–126.5)	0.000294	0.00536
*BMPR1A*	NM_004329.2:c.1348G>A	1.261%	0.075%	0.042%	17.0 (1.9–204.3)	0.00476	0.0606	30.7 (5.2–128.4)	0.000276	0.00536
*IL21R*	NM_021798.4:c.305C>T	0.840%	0.000%	0.005%	Inf (2.1–Inf)	0.00665	0.0606	162.3 (8.4–8835.8)	0.000442	0.00725
*N4BP2*	NM_018177.5:c.2582A>T	0.840%	0.000%	0.010%	Inf (2.1–Inf)	0.00665	0.0606	81.3 (5.9–1093.6)	0.000877	0.0131
*FLNA*	NM_001110556.2:c.2876G>A	1.681%	0.112%	0.164%	15.2 (2.6–104.4)	0.00125	0.0606	10.4 (2.6–30.7)	0.000989	0.0135
*KMT2C*	NM_170606.2:c.9530G>A	0.840%	0.000%	0.016%	Inf (2.1–Inf)	0.00665	0.0606	54.2 (4.5–482.3)	0.00145	0.0171
*ZEB1*	NM_001323674.1:c.2321C>T	0.840%	0.000%	0.016%	Inf (2.1–Inf)	0.00665	0.0606	54.1 (4.5–481.3)	0.00146	0.0171
*FAT4*	NM_001291285.1:c.10310C>A	0.840%	0.000%	0.031%	Inf (2.1–Inf)	0.00665	0.0606	27.1 (2.7–152.4)	0.00397	0.0367
*FANCE*	NM_021922.2:c.1333C>T	0.840%	0.000%	0.042%	Inf (2.1–Inf)	0.00665	0.0606	20.4 (2.1–102.6)	0.00626	0.0461
*MPL*	NM_005373.2:c.1300G>A	0.840%	0.000%	0.047%	Inf (2.1–Inf)	0.00665	0.0606	18.1 (1.9–88.2)	0.00760	0.0477
*LRP1B*	NM_018557.2:c.5837A>T	0.840%	0.000%	0.047%	Inf (2.1–Inf)	0.00665	0.0606	18.1 (1.9–88.0)	0.00762	0.0477
*NIN*	NM_020921.3:c.2867G>A	0.840%	0.000%	0.047%	Inf (2.1–Inf)	0.00665	0.0606	18.1 (1.9–88.2)	0.00759	0.0477
*NKX2-1*	NM_003317.4:c.251G>A	0.840%	0.000%	0.054%	Inf (2.1–Inf)	0.00665	0.0606	15.7 (1.7–74.3)	0.0097	0.0497
*CD28*	NM_001243078.1:c.298C>T	0.840%	0.000%	0.088%	Inf (2.1–Inf)	0.00665	0.0606	9.6 (1.1–40.7)	0.0222	0.0865

**Table 2:** Allele frequencies and case-control association analysis of 17 selected variants in the validation cohort.

Gene	Variant	RefSNP	Allele Frequency in Validation Cohort (n = 156)	Allele Frequency in SG10K_Health (n = 9770)	Odds Ratio (5–95% CI)	*p*-Value
*JAK2*	NM_004972.3:c.1174G>A	rs200018153	5/312 (1.603%)	180/19178 (0.939%)	1.7 (0.5–4.1)	0.226
*APOB*	NM_000384.3:c.6698T>C	rs1176839033	0/312 (0.000%)	N/A	N/A	N/A
*FAT3*	NM_001008781.2:c.8983C>A	rs62622785	0/312 (0.000%)	109/19068 (0.572%)	0.0 (0.0–2.1)	0.426
*PRDM16*	NM_022114.4:c.2062G>A	rs367580261	1/312 (0.321%)	40/18760 (0.213%)	1.5 (0.0–8.9)	0.492
*BMPR1A*	NM_004329.2:c.1348G>A	rs55932635	0/312 (0.000%)	5/19048 (0.026%)	0.0 (0.0–66.8)	1.000
*IL21R*	NM_021798.4:c.305C>T	rs1180106880	0/312 (0.000%)	16/18962 (0.084%)	0.0 (0.0–15.8)	1.000
*N4BP2*	NM_018177.5:c.2582A>T	rs61748748	0/312 (0.000%)	173/19110 (0.905%)	0.0 (0.0–1.3)	0.121
*FLNA*	NM_001110556.2:c.2876G>A	rs782104597	0/312 (0.000%)	14/13534 (0.103%)	0.0 (0.0–13.1)	1.000
*KMT2C*	NM_170606.2:c.9530G>A	rs535118581	0/312 (0.000%)	5/19106 (0.026%)	0.0 (0.0–67.0)	1.000
*ZEB1*	NM_001323674.1:c.2321C>T	rs759615272	0/312 (0.000%)	N/A	N/A	N/A
*FAT4*	NM_001291285.1:c.10310C>A	rs761789494	0/312 (0.000%)	5/19210 (0.026%)	0.0 (0.0–67.4)	1.000
*FANCE*	NM_021922.2:c.1333C>T	rs141551053	0/312 (0.000%)	16/18978 (0.084%)	0.0 (0.0–15.8)	1.000
*MPL*	NM_005373.2:c.1300G>A	rs754296556	0/312 (0.000%)	5/19024 (0.026%)	0.0 (0.0–66.8)	1.000
*LRP1B*	NM_018557.2:c.5837A>T	rs776616686	1/312 (0.321%)	27/19236 (0.140%)	2.3 (0.1–14.0)	0.363
*NIN*	NM_020921.3:c.2867G>A	rs199887033	2/312 (0.641%)	60/19098 (0.314%)	2.0 (0.2–7.8)	0.263
*NKX2-1*	NM_003317.4:c.251G>A	rs757703309	1/312 (0.321%)	5/18862 (0.027%)	12.1 (0.3–109.0)	0.0938
*CD28*	NM_001243078.1:c.298C>T	rs200936737	0/312 (0.000%)	11/18986 (0.058%)	0.0 (0.0–24.3)	1.000

N/A—These variants were not reported in the SG10K_Health cohort, so their allele frequency was unavailable, and the statistical tests were not done.

**Table 3:** Gene-based burden testing of 17 prioritised candidate genes.

Gene	Protein Function ^a^	Cases with Rare Pathogenic Variants (n = 119)	Controls with Rare Pathogenic Variants (n = 1337)	*p*-Value	FDR Adjusted *p*-Value	β Estimate	β Standard Error
*JAK2*	Cytokine and growth factor signalling	9/119 (7.56%)	32/1337 (2.39%)	0.0209	0.998	0.07955	0.03441
*APOB*	LDL receptor ligand	8/119 (6.72%)	46/1337 (3.44%)	0.134	0.998	0.04427	0.02949
*FAT3*	Cell-cell adhesion (predicted)	11/119 (9.24%)	101/1337 (7.55%)	0.697	0.998	0.00824	0.02113
*PRDM16*	Transcription factor; involved in some MDS/AML translocations	5/119 (4.20%)	42/1337 (3.14%)	0.728	0.998	0.01111	0.03198
*BMPR1A*	Type I transmembrane serine/threonine receptor	5/119 (4.20%)	7/1337 (0.52%)	0.00299	0.468	0.18300	0.06152
*IL21R*	Cell growth; proliferation and differentiation of T cells, B cells, and NK cells	2/119 (1.68%)	2/1337 (0.15%)	0.00978	0.674	0.27530	0.10640
*N4BP2*	Ubiquitylation substrate (predicted); transcription-coupled DNA repair (predicted); recombination (predicted)	2/119 (1.68%)	33/1337 (2.47%)	0.649	0.998	-0.01689	0.03709
*FLNA*	Cytoskeleton structure and remodelling	3/119 (2.52%)	41/1337 (3.07%)	0.831	0.998	−0.00699	0.03269
*KMT2C*	Histone methylation and transcriptional coactivation	6/119 (5.04%)	56/1337 (4.19%)	0.990	0.998	−0.00037	0.02824
*ZEB1*	Transcription repressor of IL-2 (predicted)	2/119 (1.68%)	12/1337 (0.90%)	0.347	0.998	0.06365	0.06773
*FAT4*	Regulating of planar cell polarity	16/119 (13.45%)	121/1337 (9.05%)	0.154	0.998	0.02757	0.01935
*FANCE*	DNA cross-link repair	3/119 (2.52%)	8/1337 (0.60%)	0.120	0.998	0.10020	0.06451
*MPL*	Regulator of megakaryopoiesis and platelet production	0/119 (0.00%)	10/1337 (0.75%)	0.507	0.998	−0.04494	0.06773
*LRP1B*	LDL receptor	10/119 (8.40%)	107/1337 (8.00%)	0.850	0.998	−0.00388	0.02053
*NIN*	Critical for centrosome function	7/119 (5.88%)	68/1337 (5.09%)	0.855	0.998	0.00464	0.02534
*NKX2-1*	Homeobox regulating morphogenesis and thyroid-specific genes	2/119 (1.68%)	0/1337 (0.00%)	0.0000184	0.0144	0.64450	0.15000
*CD28*	T-cell proliferation and survival; cytokine production	3/119 (2.52%)	1/1337 (0.07%)	0.000775	0.202	0.35340	0.10490

^a^ Obtained via RefGene.

**Table 4:** Gene-based burden testing of 17 prioritised candidate genes in 16 probands and 38 family members (2 affected, 36 unaffected).

Gene	Cases with Pathogenic Variants (n = 18)	Controls with Pathogenic Variants (n = 36)	*p*-Value	β Estimate	β Standard Error
*JAK2*	0 (0.00%)	1 (2.78%)	0.435	−0.37660	0.47920
*APOB*	18 (100.00%)	36 (100.00%)	0.625	−0.06977	0.14200
*FAT3*	4 (22.22%)	8 (22.22%)	0.907	0.01852	0.15700
*PRDM16*	2 (11.11%)	6 (16.67%)	0.677	−0.07689	0.18360
*BMPR1A*	1 (5.56%)	2 (5.56%)	0.986	−0.00504	0.28290
*IL21R*	0 (0.00%)	0 (0.00%)	N/A	N/A	N/A
*N4BP2*	1 (5.56%)	1 (2.78%)	0.646	0.15840	0.34280
*FLNA*	1 (5.56%)	4 (11.11%)	0.498	−0.15180	0.22260
*KMT2C*	18 (100.00%)	36 (100.00%)	N/A	N/A	N/A
*ZEB1*	0 (0.00%)	0 (0.00%)	N/A	N/A	N/A
*FAT4*	15 (83.33%)	34 (94.44%)	0.218	−0.27570	0.22130
*FANCE*	2 (11.11%)	0 (0.00%)	0.0376	0.70240	0.32920
*MPL*	0 (0.00%)	0 (0.00%)	N/A	N/A	N/A
*LRP1B*	1 (5.56%)	2 (5.56%)	0.978	0.00781	0.28300
*NIN*	13 (72.22%)	26 (72.22%)	0.833	0.03131	0.14810
*NKX2-1*	0 (0.00%)	0 (0.00%)	N/A	N/A	N/A
*CD28*	0 (0.00%)	0 (0.00%)	N/A	N/A	N/A

N/A—No pathogenic variants were found in these genes for both affected and unaffected individuals, so no statistical test was performed.

**Table 5:** Top 10 canonical pathways identified by IPA Pathway Analysis.

Top Canonical Pathways	IPA Enrichment *p*-Value ^a^	IPA Overlap ^a^	Individuals with Variants in Pathway Genes		Odds Ratio (95% CI) ^b^	Odds Ratio *p*-Value ^b^	Gene
			Cases	Controls			
GM-CSF Signalling	0.0092	6/70 (8.6%)	17/119 (14.29%)	41/1337 (3.07%)	5.3 (2.7–9.9)	1.21 × 10^−6^	*CAMK2D, CSF2RA, GRB2, JAK2, PPP3CC, RALB*
Synaptogenesis Signalling Pathway	0.0158	15/312 (4.8%)	29/119 (24.37%)	61/1337 (4.56%)	6.7 (4.0–11.2)	6.11 × 10^−12^	*ACTR3, AP2A2, CACNB2, CAMK2D, CDH8, CREB1, CTNNB1, EFNA2, GRB2, NAP1L1, NSF, RAB5B, RALB, SHF, SYT7*
Clathrin-mediated Endocytosis Signalling	0.0274	10/192 (5.2%)	19/119 (15.97%)	25/1337 (1.87%)	9.9 (5.0–19.5)	1.35 × 10^−10^	*ACTA1, ACTR3, AP2A2, EPS15, GRB2, IGF1, ORM1, ORM2, PPP3CC, RAB5B*
Role of JAK1 and JAK3 in γc Cytokine Signalling	0.0291	5/67 (7.5%)	16/119 (13.45%)	41/1337 (3.07%)	4.9 (2.5–9.3)	4.87 × 10^−6^	*GRB2, IL15, IL21R, JAK2, RALB*
Agrin Interactions at Neuromuscular Junction	0.0307	5/68 (7.4%)	29/119 (24.37%)	127/1337 (9.50%)	3.1 (1.9–4.9)	7.13 × 10^−6^	*ACTA1, LAMA2, NRG3, PAK4, RALB*
NAD biosynthesis II (from tryptophan)	0.0312	2/11 (18%)	5/119 (4.20%)	6/1337 (0.45%)	9.7 (2.3–38.8)	1.04 × 10^−3^	*AFMID, NMNAT3*
Tryptophan Degradation III (Eukaryotic)	0.0314	3/27 (11%)	9/119 (7.56%)	22/1337 (1.65%)	4.9 (1.9–11.4)	5.20 × 10^−4^	*AFMID, HADHA, L3HYPDH*
CREB Signalling in Neurons	0.0355	23/595 (3.9%)	57/119 (47.90%)	160/1337 (11.97%)	6.7 (4.5–10.2)	1.61 × 10^−19^	*ADGRB1, ADORA2B, ADRA1A, BMPR1A, BMPR1B, CACNB2, CAMK2D, CREB1, DRD1, DRD3, FFAR2, GHRHR, GLP2R, GPR108, GPR27, GPR63, GRB2, IGF1, PDGFRA, PTGER1, RALB, RXFP1, SSTR3*
IL-15 Production	0.0357	7/120 (5.8%)	30/119 (25.21%)	88/1337 (6.58%)	4.8 (2.9–7.8)	1.98 × 10^−9^	*CSF2RA, DSTYK, IL15, JAK2, PDGFRA, TNK1, ZAP70*
Caveolar-mediated Endocytosis Signalling	0.0441	5/75 (6.7%)	6/119 (5.04%)	1/1337 (0.07%)	70.4 (8.4–3197.9)	1.73 × 10^−6^	*ACTA1, CAVIN1, FLOT2, HLA-E, RAB5B*

^a^ IPA enrichment *p*-value and IPA overlap tests for over-represented biological pathways in the list of genes with significantly different germline mutation burden in cases as compared to controls. The IPA enrichment *p*-values were calculated using Fisher’s exact test. IPA overlap represents the number of genes in our dataset over the total number of genes that make up the pathway in the Ingenuity Knowledge Base. ^b^ Odds ratio and odds ratio *p*-value tests if case or control individuals are over-represented in the list of individuals with any rare pathogenic variant in each pathway.

## Data Availability

A publicly available dataset, gnomAD, was analyzed in this study and can be accessed at https://gnomad.broadinstitute.org/ (accessed on 1 June 2022). Restrictions apply to the availability of data from SG10K_Health, which can be requested from the Singapore National Precision Medicine program. Gene expression data from TCGA can be accessed at https://portal.gdc.cancer.gov/ (accessed on 1 June 2022).

## Supplementary Materials

The following supporting information can be downloaded at: https://www.mdpi.com/article/10.3390/cancers14153680/s1, Figure S1: Principal component analysis (PCA) plot of germline genotypes for the discovery and local control cohorts, with genotypes from the 1000 Genomes Project (1KGP) as reference; Figure S2: QQ-plot *R*^2^ values for different read depth cut-offs and number of PCA covariates; Figure S3: Representative IGV alignments of problematic variants manually removed via IGV checks; Figure S4: Oncoplot of 17 variants in 17 prioritized candidate genes, showing the frequency of each variant; Figure S5: Gene expression for six result genes in primary tumors versus normal tissue in six cancers; Figure S6: Top 10 canonical pathways identified by IPA Pathway Analysis; Table S1: Demographic and family history characteristics of NPC patients in the discovery and validation cohorts; Table S2: Table of rare predicted pathogenic variants in prioritised genes found only in single patients; Table S3: Table of 17 prioritised genes with variants in the NPC discovery cohort (n = 119), annotated with supporting information from cancer gene databases; Table S4: Pathogenicity of 17 variants in 17 known or candidate cancer genes using in silico prediction tools and database classifications; Table S5: Variants associated with NPC in the prior literature; Table S6: Genes associated with NPC in the prior literature.
