# Supplementary material for: Germline Variants Associated with Nasopharyngeal Carcinoma Predisposition Identified through Whole-Exome Sequencing
Source: Cancers (Basel). 2022 Jul 28;14(15):3680. doi: 10.3390/cancers14153680 (PMC9367457; doi:10.3390/cancers14153680)
Supplement: Supplementary file 1 [file cancers-14-03680-s001.zip › cancers-1816244-supplementary.pdf]

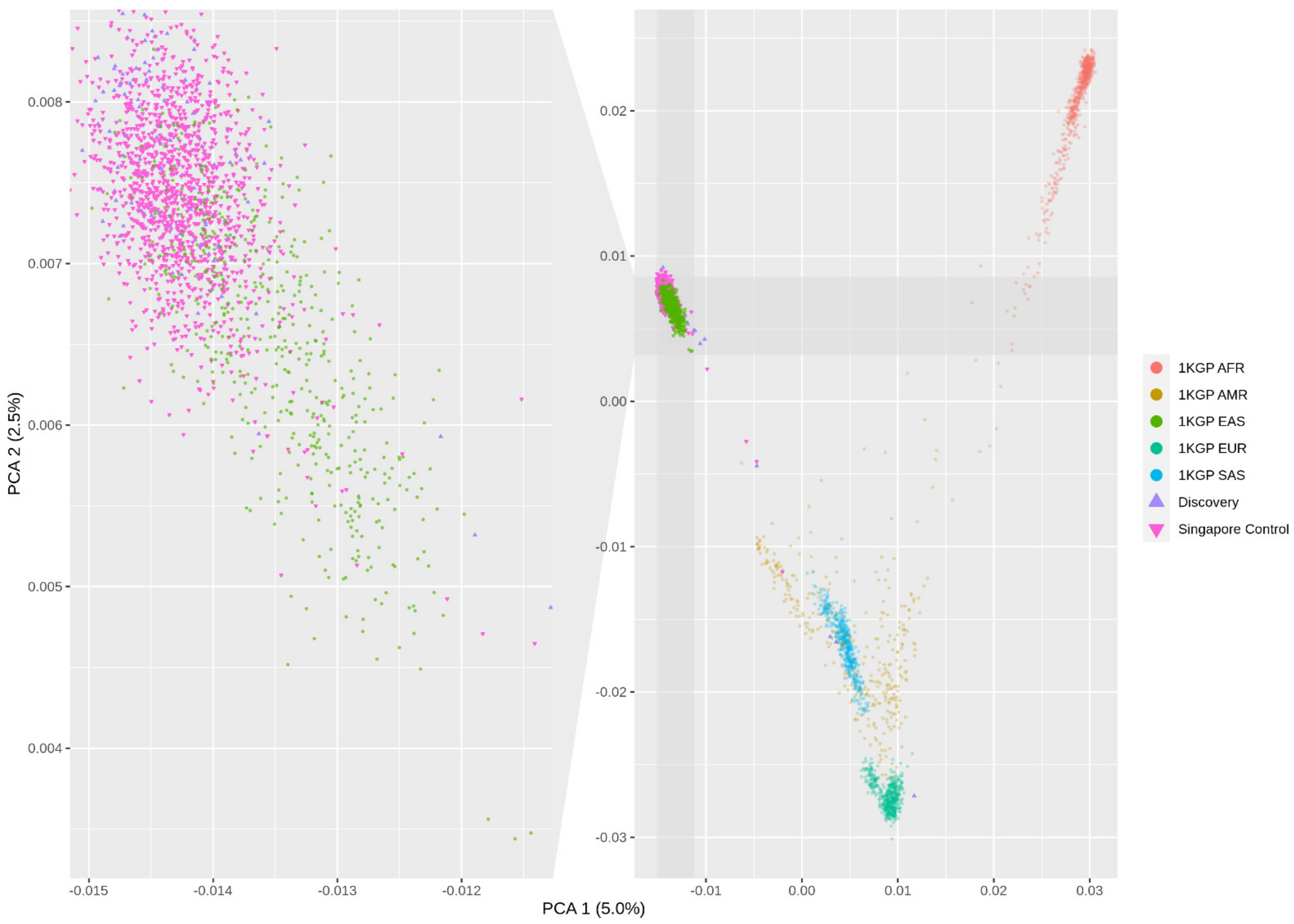

Supplementary Figure S1. Principal component analysis (PCA) plot of germline genotypes for the discovery and local control cohorts, with genotypes from the 1000 Genomes Project (1KGP) as reference.

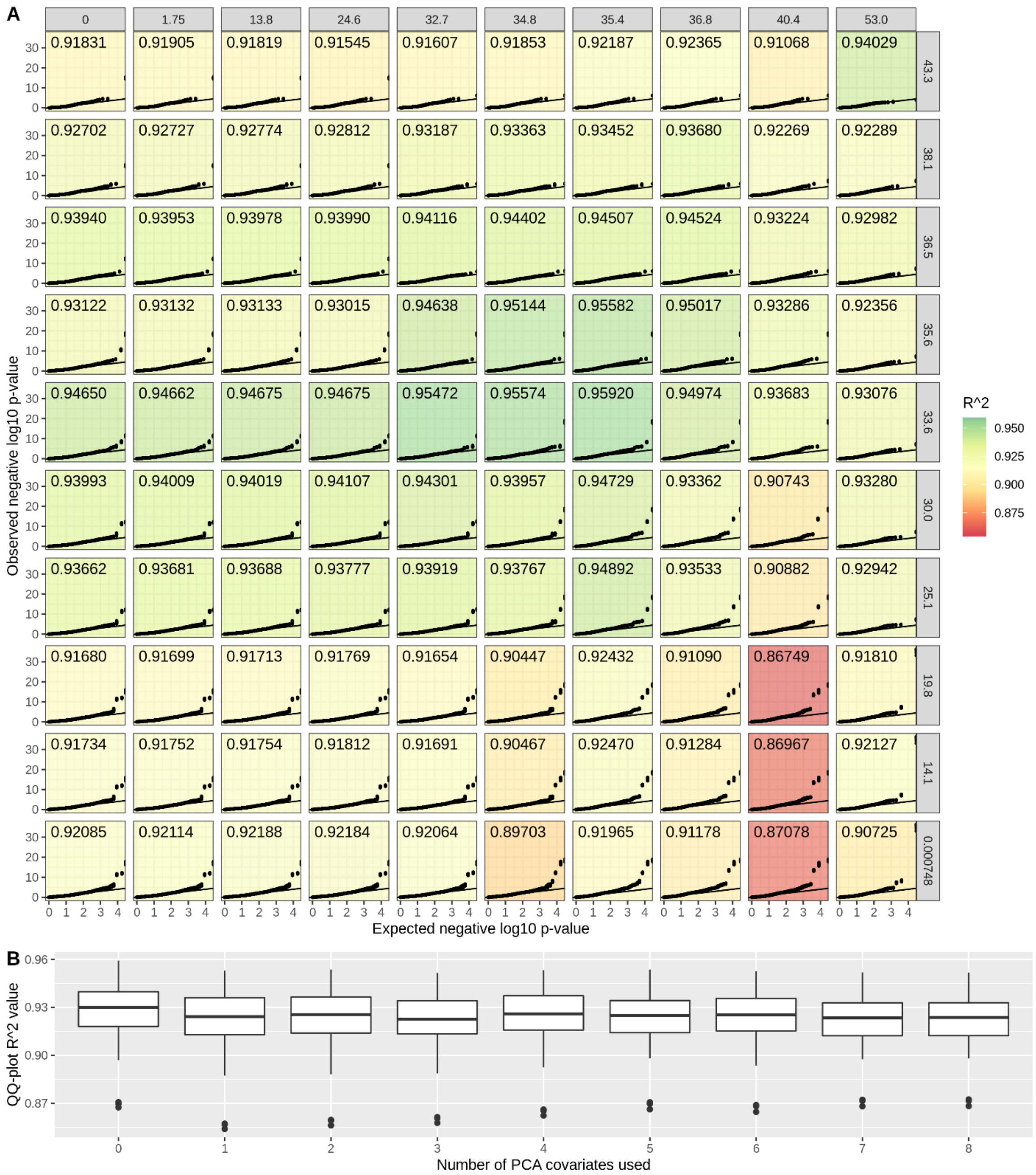

Supplementary Figure S2. QQ-plot R2 values for different read depth cut-offs and number of PCA covariates. (A) QQ-plots and their respective R^2 values for a gene-based burden test on rare synonymous variants, without using any PCA covariates. Each column represents one value of average read depth cut-off for the case cohort; each row represents one value for the local control cohort. (B) Box plots of QQ-plot R^2 values for different numbers of PCA covariates used. Each box represents 100 R^2 values for each combination of case and local control average read depth cut-offs.

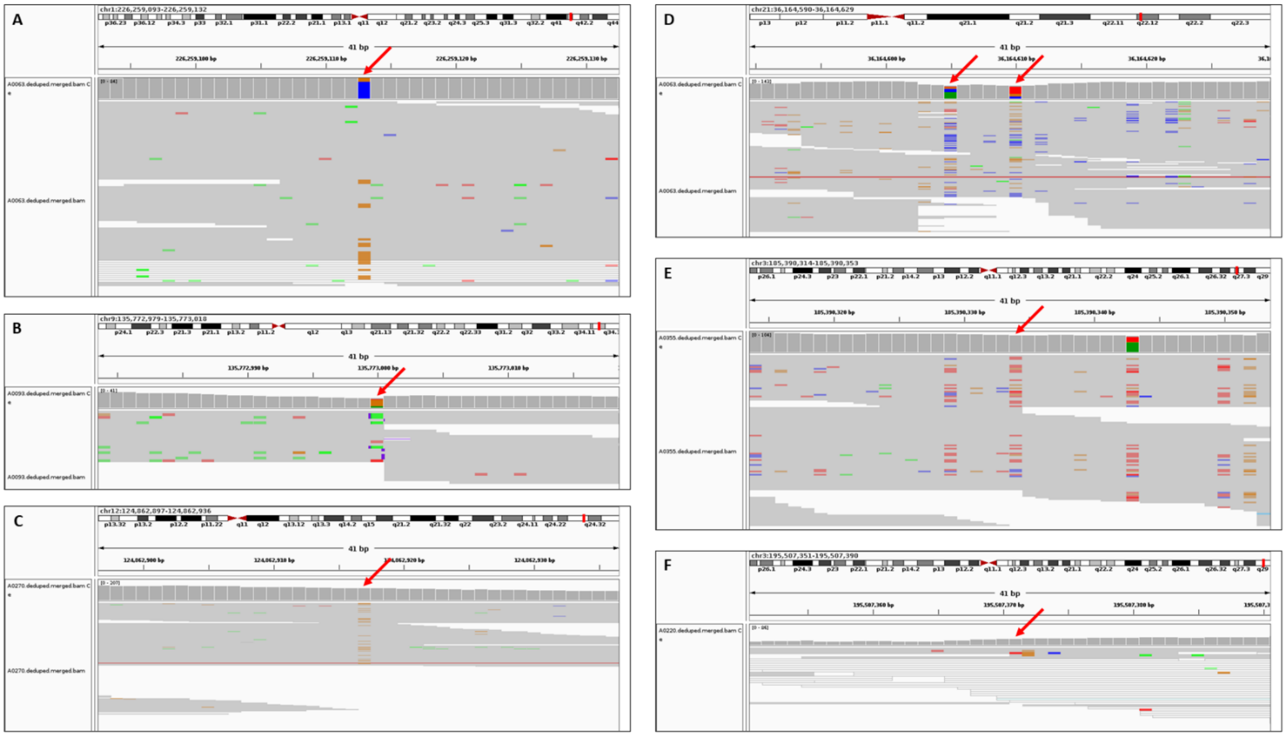

Supplementary Figure S3. Representative IGV alignments of problematic variants manually removed via IGV checks. These include (A) a H3F3A nonsynonymous SNV (rs749423281) with multiple mismatches; (B) a rarely called TSC1 insertion (rs760737807) adjacent to a common misalignment indel; (C) a NCOR2 nonsynonymous SNV (rs2040173313) with low variant allele frequency; (D) two RUNX1 nonsynonymous SNVs (rs2056451534 and rs2056451758) with read support for multiple alternative alleles; (E) a IGF2BP2 nonsynonymous SNV (rs1718679984) in a high discrepancy region; and (F) a MUC4 frameshift deletion (rs771863899) with low mapping quality. The position of each problematic variant is indicated by the red arrows.

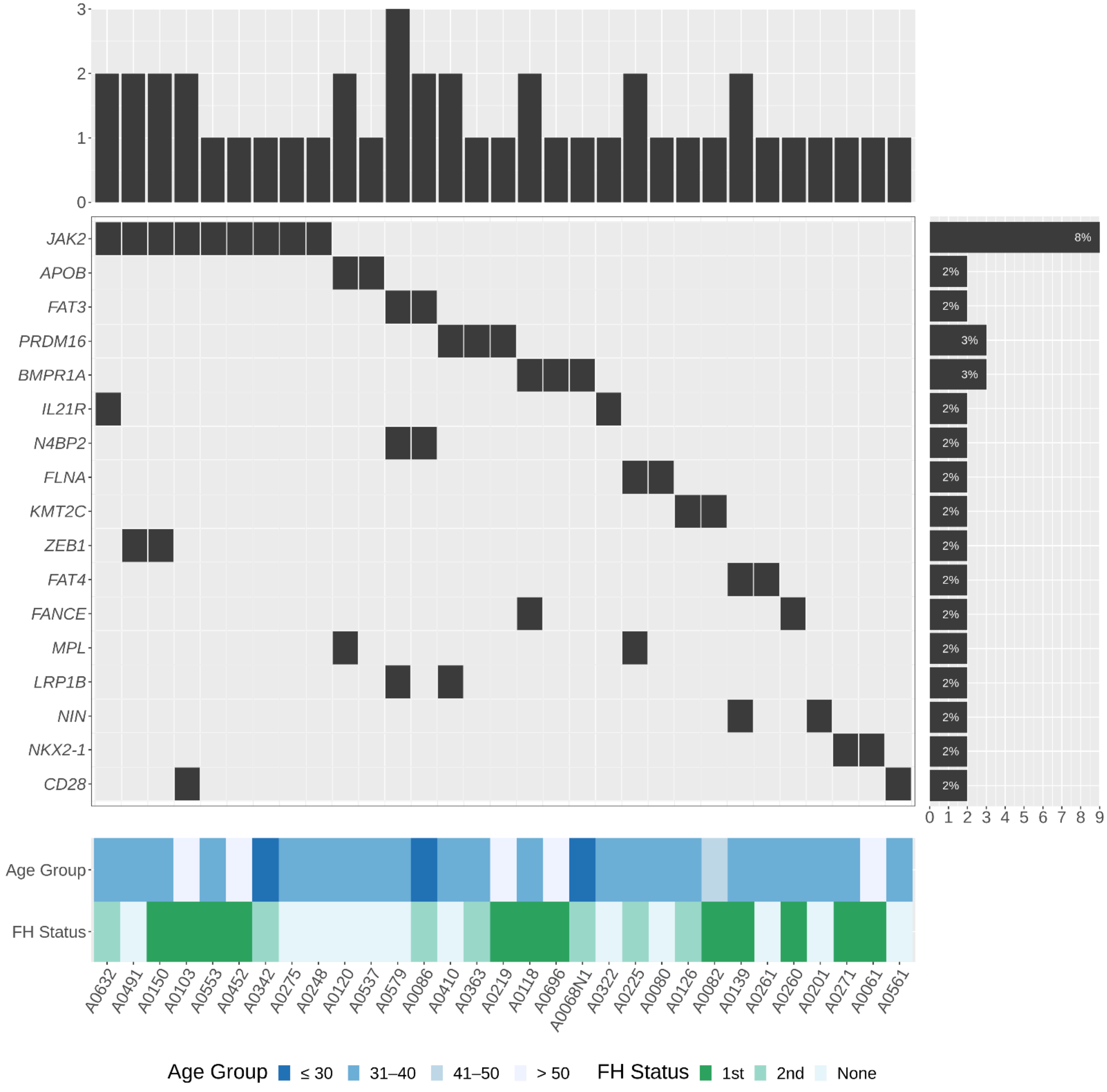

Supplementary Figure S4. Oncoplot of 17 variants in 17 prioritized candidate genes, showing the frequency of each variant. Rows represent genes and each column represents one case. Rows (bottom) show the age group and family history (FH) status for each case.

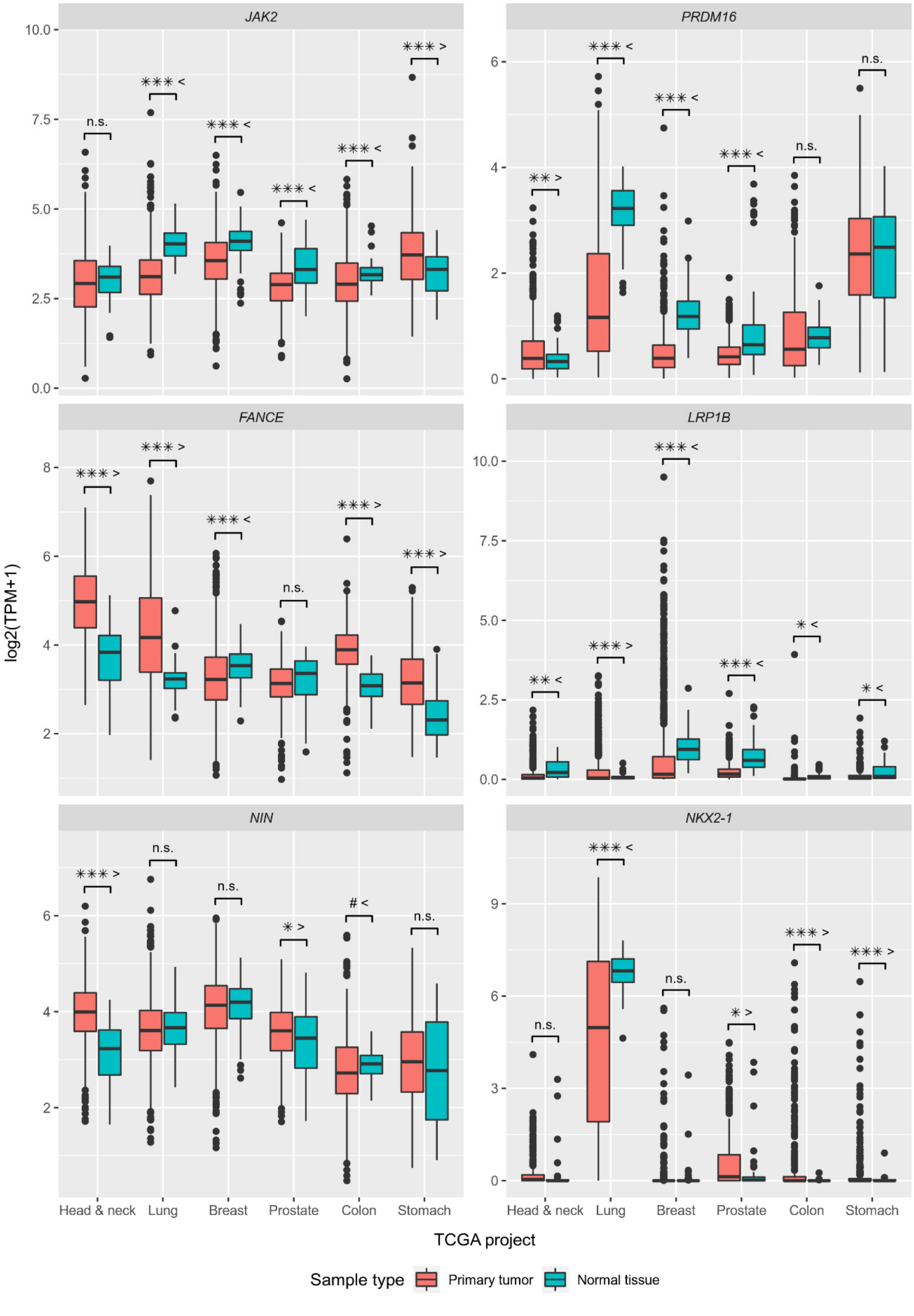

Supplementary Figure S5. Gene expression for six genes in primary tumors versus normal tissue, for six cancers. The expression data was obtained from TCGA projects HNSC, LUAD plus LUSC, BRCA, PRAD, COAD, and STAD respectively; *p*-values were obtained from a two-tailed t-test of log(TPM+1) primary tumor versus normal tissue. Significance codes: "***" if $p < 0.001$; "**" if $p < 0.01$; "*" if $p < 0.05$; "#" if $p < 0.1$; and "n.s." for not significant. A greater-than symbol (">") after a significance code indicates higher expression in primary tumor; a less-than symbol ("<") indicates higher expression in normal tissue.

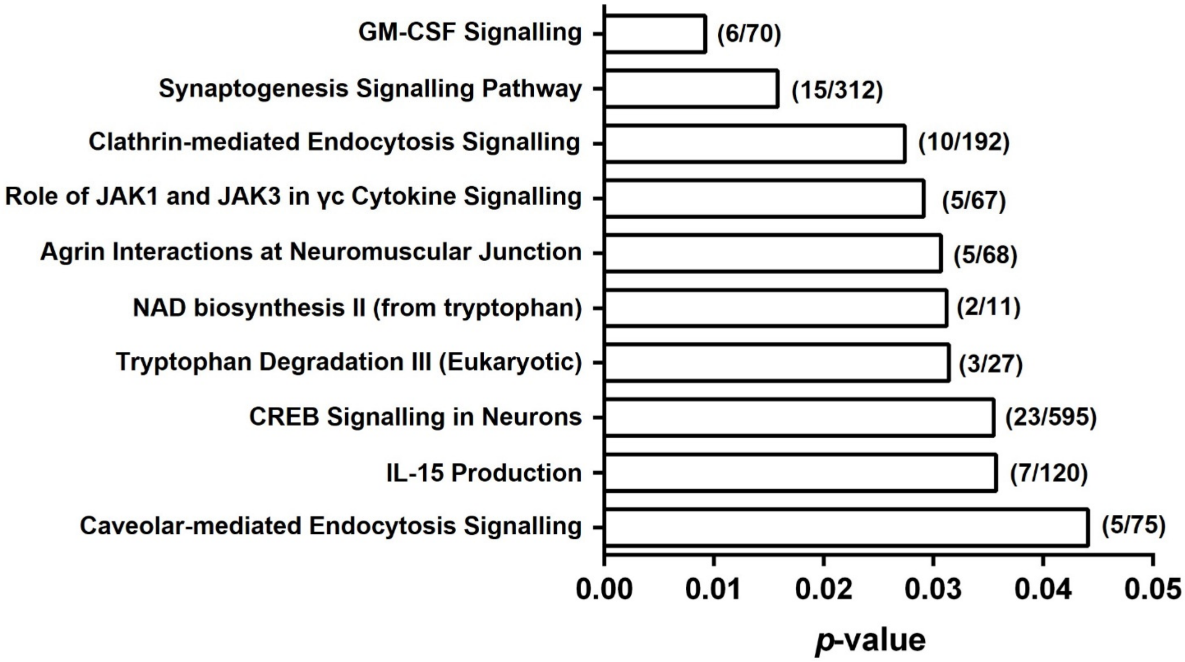

Supplementary Figure S6. Top 10 canonical pathways identified by IPA Pathway Analysis. Bar graphs are sorted by the rank of *p*-value in an increasing order. Numbers in parentheses refer to the number of genes in our dataset over the total number of genes that make up the canonical pathway in the Ingenuity Knowledge Base.

Supplementary Table S1. Demographic and family history characteristics of NPC patients in the discovery and validation cohorts.

Characteristics	Discovery cohort (n=119)						Validation cohort (n=156)	
	Young patients, age ≤40 years at diagnosis (n=90)		Older patients with family history, age >40 years at diagnosis (n=29)		Overall			
	No. of participants	%	No. of participants	%	No. of participants	%	No. of participants	%
**Age at diagnosis, years**								
Mean ± S.D	35.09 ± 5.24	-	55.31 ± 7.03	-	40.02 ± 10.41	-	34.02 ± 4.97	-
Range (min - max)	13 - 40	-	43 - 68	-	13 - 68	-	17 - 40	-
**Gender**								
Male	64	71.11	23	79.31	87	73.11	104	66.67
Female	26	28.89	6	20.69	32	26.89	52	33.33
**Race**								
Chinese	79	87.78	26	89.66	105	88.24	132	84.62
Indian	0	0	1	3.45	1	0.84	0	0
Malay	2	2.22	1	3.45	3	2.52	9	5.77
Others	9	10	1	3.45	10	8.4	15	9.62
**Family History**								
1st degree	20	22.22	27	93.1	47	39.5	14	8.97
2nd degree	17	18.89	2	6.9	19	15.97	10	6.41
Unknown	-	-	-	-	-	-	16	10.26
None	53	58.89	0	0	53	44.54	116	74.36

Supplementary Table S2. Table of rare predicted pathogenic variants in prioritised genes found only in single patients.

Supplementary Table S3. Table of 17 prioritised genes with variants in the NPC discovery cohort (n=119), annotated with supporting information from cancer gene databases.

Gene	Number of cases with the significant variant	NCG 6.0 [a]			COSMIC CGC v94		Literature support		
		Known (n=711)	Candidates by primary site (n=104)	Candidates by number of tumours (n=711)	Germline (n=107)	Somatic (n=680)	Cancer predisposition genes [b] (n=114)	Driver genes [c] (n=299)	Driver Genes (nucleotide context) [d] (n=460)
JAK2	9 (7.56%)	Y				Y		Y	
APOB	2 (1.68%)		Y	Y				Y	
FAT3	2 (1.68%)	Y				Y			Level A
PRDM16	3 (2.52%)	Y				Y			
BMPR1A	3 (2.52%)	Y			Y		Y		
IL21R	2 (1.68%)	Y				Y			
N4BP2	2 (1.68%)	Y				Y			
FLNA	2 (1.68%)	Y				Y		Y	
KMT2C	2 (1.68%)	Y				Y		Y	Level A
ZEB1	2 (1.68%)	Y				Y			
FAT4	2 (1.68%)	Y				Y			Level A
FANCE	2 (1.68%)	Y			Y				
MPL	2 (1.68%)	Y			Y	Y			
LRP1B	2 (1.68%)	Y				Y			
NIN	2 (1.68%)	Y				Y			
NKX2-1	2 (1.68%)	Y				Y			
CD28	2 (1.68%)	Y				Y			

Y – Gene is present in the given database.

[a] – Based on the NCG downloadable list of "known cancer genes," and lists of "candidate cancer genes with strong support" [32].

[b] – Based on a list of cancer predisposition genes [17].

[c] – Based on a curated list of cancer driver genes [34].

[d] – Based on a curated list of cancer driver genes based on nucleotide context [35]. Levels range from A (highest) to D (lowest).

Supplementary Table S4. Pathogenicity of 17 variants in 17 known or candidate cancer genes using in silico prediction tools and database classifications.

Gene	Variant	Consequence	Protein Alteration	Transcript	SIFT Prediction	PolyPhen2 Prediction	Mutation Taster Prediction	CADD Score	ClinVar Classification	InterVar Classification	SampleID
JAK2	c.1174G>A	Nonsynonymous SNV	p.V392M	NM_004972.3	Deleterious	Probably damaging	Polymorphism	22.6	Likely benign	Uncertain significance	A0103, A0150, A0248, A0275, A0342, A0452, A0491, A0553, A0632
APOB	c.6698T>C	Nonsynonymous SNV	p.I2233T	NM_000384.3	Deleterious	Possibly damaging	Disease causing	21.0	Uncertain significance	Uncertain significance	A0120, A0537
FAT3	c.8983C>A	Nonsynonymous SNV	p.Q2995K	NM_001008781.2	Tolerated	Benign	Disease causing	23.3	N/A	Uncertain significance	A0086, A0579
PRDM16	c.2062G>A	Nonsynonymous SNV	p.A688T	NM_022114.4	Deleterious	Probably damaging	Disease causing	20.6	Uncertain significance	Uncertain significance	A0219, A0363, A0410
BMPR1A	c.1348G>A	Nonsynonymous SNV	p.V450M	NM_004329.2	Tolerated	Benign	Disease causing	22.1	Conflicting interpretations of pathogenicity	Uncertain significance	A0068N1, A0118, A0696
IL21R	c.305C>T	Nonsynonymous SNV	p.S102F	NM_021798.4	Deleterious	Benign	Polymorphism	22.4	N/A	Uncertain significance	A0322, A0632
N4BP2	c.2582A>T	Nonsynonymous SNV	p.E861V	NM_018177.5	Deleterious	Probably damaging	Disease causing	22.8	N/A	Uncertain significance	A0086, A0579
FLNA	c.2876G>A	Nonsynonymous SNV	p.S959N	NM_001110556.2	Deleterious	Probably damaging	Disease causing	29.4	N/A	Uncertain significance	A0080, A0225
KMT2C	c.9530G>A	Nonsynonymous SNV	p.R3177H	NM_170606.2	Deleterious	Probably damaging	Disease causing	31	N/A	Uncertain significance	A0082, A0126
ZEB1	c.2321C>T	Nonsynonymous SNV	p.T774M	NM_001323674.1	Deleterious	Probably damaging	Disease causing	24.2	N/A	Uncertain significance	A0150, A0491
FAT4	c.10310C>A	Nonsynonymous SNV	p.P3437H	NM_001291285.1	Deleterious	Probably damaging	Disease causing	26.2	N/A	Uncertain significance	A0261, A0139
FANCE	c.1333C>T	Nonsynonymous SNV	p.P445S	NM_021922.2	Tolerated	Benign	Polymorphism	20.3	Conflicting interpretations of pathogenicity	Likely benign	A0118, A0260
MPL	c.1300G>A	Nonsynonymous SNV	p.D434N	NM_005373.2	Tolerated	Probably damaging	Polymorphism	23.6	N/A	Uncertain significance	A0225, A0120
LRP1B	c.5837A>T	Nonsynonymous SNV	p.D1946V	NM_018557.2	Tolerated	Probably damaging	Disease causing	23.0	N/A	Uncertain significance	A0410, A0579
NIN	c.2867G>A	Nonsynonymous SNV	p.C956Y	NM_020921.3	Deleterious	Probably damaging	Disease causing	21.6	N/A	Uncertain significance	A0139, A0201
NKX2-1	c.251G>A	Nonsynonymous SNV	p.G84E	NM_003317.4	Deleterious	Probably damaging	Disease causing	27.6	N/A	Uncertain significance	A0061, A0271
CD28	c.298C>T	Nonsynonymous SNV	p.R100C	NM_001243078.1	Deleterious	Probably damaging	Disease causing	35.0	N/A	Uncertain significance	A0103, A0561

N/A Not available. These variants are not represented in the ClinVar database, and have no available ClinVar classification.

Supplementary Table S5. Variants associated with NPC in prior literature.

Study	Gene	RefSNP ID	Chrom.	GRCh37 position	Ref allele	Risk allele	Region	CADD	Discovery (n=119)	Local controls (n=1,337)			gnomAD NC (EAS) (n=9,626)		
									Allele frequency	Allele frequency	Odds ratio (95% CI)	*p*-value	Allele frequency	Odds ratio (95% CI)	*p*-value
Yu et al. (2019)	*BRD2*	rs778675391	chr6	32948153	A	T	exonic	23.2	7/238 (2.941%)	13/2674 (0.486%)	6.2 (2.1–16.9)	6.85E-04	75/19242 (0.390%)	7.7 (3.0–17.0)	6.49E-05
Tang et al. (2012)	*GABBR1*	rs2267633	chr6	29570841	A	G	UTR3	1.1	40/238 (16.807%)	710/2674 (26.552%)	0.6 (0.4–0.8)	8.39E-04	2954/12036 (24.543%)	0.6 (0.4–0.9)	5.91E-03
Tse et al. (2009)	*GABBR1*	rs2267633	chr6	29570841	A	A	UTR3	-	198/238 (83.193%)	1964/2674 (73.448%)	1.8 (1.3–2.6)	8.39E-04	9082/12036 (75.457%)	1.6 (1.1–2.3)	5.91E-03
Tse et al. (2009)	*GABBR1*	rs29230	chr6	29576393	A	A	exonic	-	198/238 (83.193%)	1966/2674 (73.523%)	1.8 (1.2–2.6)	8.39E-04	14538/19232 (75.593%)	1.6 (1.1–2.3)	6.07E-03
Tse et al. (2009)	*GABBR1*	rs2076483	chr6	29571545	A	A	intronic	-	198/238 (83.193%)	1985/2674 (74.233%)	1.7 (1.2–2.5)	1.76E-03	8178/10644 (76.832%)	1.5 (1.1–2.2)	1.96E-02
Liu et al. (2019)	*BRD2*	rs76146382	chr6	32943229	T	C	exonic	0.0	24/238 (10.084%)	164/2674 (6.133%)	1.7 (1.0–2.7)	2.63E-02	975/19242 (5.067%)	2.1 (1.3–3.2)	1.58E-03
Yu et al. (2019)	*PSEN2*	rs533813519	chr1	227075798	C	A	exonic	31	1/238 (0.420%)	5/2674 (0.187%)	2.3 (0.0–20.2)	4.01E-01	53/19250 (0.275%)	1.5 (0.0–9.0)	4.85E-01
Liu et al. (2019)	*PRKDC*	rs78231671	chr8	48695175	T	C	intronic	2.4	5/238 (2.101%)	88/2674 (3.291%)	0.6 (0.2–1.6)	4.40E-01	448/12422 (3.607%)	0.6 (0.2–1.4)	2.88e-01
Ko et al. (2014)	*CCDC170*	rs3757318	chr6	151914113	G	A	intronic	0.6	67/238 (28.151%)	765/2674 (28.609%)	1.0 (0.7–1.3)	9.40E-01	439/18199 (2.412%)	15.8 (11.6–21.5)	7.25E-49
Yu et al. (2019)	*PRKDC*	rs201726098	chr8	48749063	C	T	exonic	33	0/238 (0.000%)	2/2674 (0.075%)	0.0 (0.0–60.0)	1.00E+00	8/17896 (0.045%)	0.0 (0.0–44.3)	1.00E+00
Yu et al. (2019)	*HNRNPU*	rs760669739	chr1	245021539	T	C	exonic	24.3	0/238 (0.000%)	5/2674 (0.187%)	0.0 (0.0–12.3)	1.00E+00	56/19252 (0.291%)	0.0 (0.0–5.5)	1.00E+00
Yu et al. (2019)	*RAPGEF3*	rs767963161	chr12	48143184	G	A	exonic	35	0/238 (0.000%)	1/2674 (0.037%)	0.0 (0.0–435.2)	1.00E+00	2/19250 (0.010%)	0.0 (0.0–437.0)	1.00E+00
Yu et al. (2019)	*ITGB6*	rs188076755	chr2	160993948	C	T	exonic	34	0/238 (0.000%)	1/2674 (0.037%)	0.0 (0.0–435.2)	1.00E+00	10/19233 (0.052%)	0.0 (0.0–36.3)	1.00E+00
Yu et al. (2019)	*MAML1*	rs761942273	chr5	179192654	G	C	exonic	25.6	0/238 (0.000%)	3/2674 (0.112%)	0.0 (0.0–27.3)	1.00E+00	11/19252 (0.057%)	0.0 (0.0–32.4)	1.00E+00
Guo et al. (2020)	*RPA1*	rs1131636	chr17	1801189	C	T	UTR3	7.8		1503/2674 (56.208%)			905/12040 (7.517%)		
Ko et al. (2014)	*CLPTM1L*	rs401681	chr5	1322087	C	T	intronic	3.8		842/2674 (31.488%)			505/17000 (2.971%)		

Supplementary Table S6. Genes associated with NPC in prior literature.

Studies	Gene	Cases with variants (n=119)	Local controls with variants (n=1,337)	*p*-value	FDR-adjusted *p*-value	β estimate	β standard error
Liu et al. (2019)	*BRD2*	10/119 (8.40%)	25/1337 (1.87%)	0.0002578	0.1327498	0.13730	0.03749
Wang et al. (2019)	*CTNNB1*	1/119 (0.84%)	0/1337 (0.00%)	0.0062850	0.6742400	0.58380	0.21330
Dai et al. (2016)	*TRMT10B*	1/119 (0.84%)	0/1337 (0.00%)	0.007726	0.7372183	0.56940	0.21350
Wang et al. (2019)	*IRF5*	4/119 (3.36%)	11/1337 (0.82%)	0.0393900	0.9996554	0.11540	0.05595
Wang et al. (2019)	*E2F3*	1/119 (0.84%)	1/1337 (0.07%)	0.1402000	0.9996554	0.22130	0.15000
Dai et al. (2016)	*IFRD1*	2/119 (1.68%)	7/1337 (0.52%)	0.203100	0.9996554	0.09053	0.07110
Wang et al. (2019)	*IDH1*	5/119 (4.20%)	24/1337 (1.80%)	0.2243000	0.9982000	0.04973	0.04090
Lin et al. (2014)	*KMT2D*	10/119 (8.40%)	70/1337 (5.24%)	0.236500	0.9982000	0.02947	0.02489
Li et al. (2017)	*CYLD*	0/119 (0.00%)	9/1337 (0.67%)	0.244900	0.9982000	−0.08747	0.07519
Wang et al. (2019)	*MTOR*	0/119 (0.00%)	10/1337 (0.75%)	0.2697000	0.9982000	−0.07374	0.06678
Wang et al. (2019)	*CACNA1B*	2/119 (1.68%)	45/1337 (3.37%)	0.3012000	0.9996554	−0.03252	0.03144
Wang et al. (2019)	*EGFR*	2/119 (1.68%)	14/1337 (1.05%)	0.3042000	0.9982000	0.05533	0.05384
Dai et al. (2016)	*NFXL1*	0/119 (0.00%)	15/1337 (1.12%)	0.306800	0.9996554	−0.05670	0.05546
Lin et al. (2014)	*ARID1A*	2/119 (1.68%)	41/1337 (3.07%)	0.322500	0.9982000	−0.03257	0.03291
Wang et al. (2019)	*NOTCH1*	5/119 (4.20%)	39/1337 (2.92%)	0.3335000	0.9982000	0.03247	0.03356
Wang et al. (2019)	*PTPRU*	7/119 (5.88%)	57/1337 (4.26%)	0.3361000	0.9996554	0.02648	0.02752
Dai et al. (2016)	*DEFB1*	2/119 (1.68%)	14/1337 (1.05%)	0.350500	0.9996554	0.05858	0.06272
Wang et al. (2019)	*CDC27*	0/119 (0.00%)	8/1337 (0.60%)	0.3974000	0.9996554	−0.06486	0.07662
Dai et al. (2016)	*SIRT7*	1/119 (0.84%)	4/1337 (0.30%)	0.437000	0.9996554	0.07426	0.09550
Wang et al. (2019)	*MYC*	0/119 (0.00%)	2/1337 (0.15%)	0.4393000	0.9982000	−0.11720	0.15160
Wang et al. (2019)	*KRT10*	4/119 (3.36%)	37/1337 (2.77%)	0.4673000	0.9996554	0.02617	0.03599
Wang et al. (2019)	*MAML3*	5/119 (4.20%)	41/1337 (3.07%)	0.4768000	0.9996554	0.02311	0.03248
Dai et al. (2016)	*TMC7*	1/119 (0.84%)	23/1337 (1.72%)	0.530400	0.9996554	−0.02773	0.04420
Li et al. (2017), Wang et al. (2019)	*NRAS*	0/119 (0.00%)	1/1337 (0.07%)	0.531300	0.9982000	−0.13420	0.21420
Wang et al. (2019)	*PIK3CA*	0/119 (0.00%)	16/1337 (1.20%)	0.5451000	0.9982000	−0.03228	0.05333
Dai et al. (2016)	*PCDHB13*	2/119 (1.68%)	28/1337 (2.09%)	0.551300	0.9996554	−0.02357	0.03956
Dai et al. (2016)	*ALOX12B*	0/119 (0.00%)	9/1337 (0.67%)	0.563900	0.9996554	−0.04130	0.07155
Li et al. (2017)	*MED12L*	2/119 (1.68%)	27/1337 (2.02%)	0.598800	0.9996554	−0.02148	0.04081
Liu et al. (2019)	*HNRNPU*	1/119 (0.84%)	15/1337 (1.12%)	0.7019000	0.9996554	−0.02073	0.05414
Zheng et al. (2016), Li et al. (2017)	*NFKBIA*	0/119 (0.00%)	3/1337 (0.22%)	0.711500	0.9982000	−0.04569	0.12350
Hui et al. (2005), Wang et al. (2019)	*CCND1*	0/119 (0.00%)	2/1337 (0.15%)	0.715100	0.9982000	−0.05523	0.15130
Li et al. (2017)	*TRAF3*	1/119 (0.84%)	4/1337 (0.30%)	0.725400	0.9982000	0.03375	0.09606
Li et al. (2017)	*HLA-A*	7/119 (5.88%)	84/1337 (6.28%)	0.728900	0.9982000	−0.01313	0.03787

Liu et al. (2019)	*CDKN2B*	0/119 (0.00%)	1/1337 (0.07%)	0.7541000	0.9996554	−0.06708	0.21410
Dai et al. (2016)	*GINS2*	0/119 (0.00%)	4/1337 (0.30%)	0.760500	0.9996554	−0.03247	0.10650
Lin et al. (2014)	*TET2*	2/119 (1.68%)	16/1337 (1.20%)	0.773600	0.9982000	0.01458	0.05067
Wang et al. (2019)	*ATXN1*	2/119 (1.68%)	21/1337 (1.57%)	0.7807000	0.9996554	0.01338	0.04805
Dai et al. (2016)	*ATG14*	0/119 (0.00%)	7/1337 (0.52%)	0.808900	0.9996554	−0.01962	0.08111
Lin et al. (2014) , Wang et al. (2019)	*BAP1*	1/119 (0.84%)	8/1337 (0.60%)	0.820800	0.9982000	0.01617	0.07138
Liu et al. (2019)	*CLPTM1L*	1/119 (0.84%)	7/1337 (0.52%)	0.8250000	0.9996554	−0.01674	0.07566
Dai et al. (2016)	*MST1R*	3/119 (2.52%)	39/1337 (2.92%)	0.839600	0.9996554	0.00674	0.03329
Wang et al. (2019)	*MEF2A*	0/119 (0.00%)	2/1337 (0.15%)	0.8529000	0.9996554	−0.02802	0.15110
Dai et al. (2016)	*ATG2A*	4/119 (3.36%)	36/1337 (2.69%)	0.900800	0.9996554	0.00424	0.03399
Zheng et al. (2016), Li et al. (2017), Wang et al. (2019)	*TP53*	0/119 (0.00%)	1/1337 (0.07%)	0.902500	0.9982000	−0.02609	0.21300
Wang et al. (2019)	*AKT1*	0/119 (0.00%)	3/1337 (0.22%)	0.9217000	0.9982000	0.01209	0.12300
Wang et al. (2019)	*SOX4*	0/119 (0.00%)	1/1337 (0.07%)	0.9462000	0.9996554	−0.01447	0.21420
Liu et al. (2019)	*TNFRSF19*	2/119 (1.68%)	12/1337 (0.90%)	0.9545000	0.9996554	−0.00338	0.05921
Dai et al. (2016)	*ACIN1*	3/119 (2.52%)	31/1337 (2.32%)	0.954600	0.9996554	−0.00213	0.03740
Liu et al. (2019), Wang et al. (2019)	*CDKN2A*	0/119 (0.00%)	1/1337 (0.07%)	0.9611000	0.9982000	−0.01045	0.21440
Wang et al. (2019)	*RUNX1*	1/119 (0.84%)	5/1337 (0.37%)	0.9674000	0.9982000	−0.00358	0.08752
Lin et al. (2014)	*KMT2C*	6/119 (5.04%)	56/1337 (4.19%)	0.989700	0.9982000	−0.00037	0.02824
Wang et al. (2019)	*LTBR*	0/119 (0.00%)	1/1337 (0.07%)	0.9999000	0.9999000	0.00003	0.21360
Dai et al. (2016)	*ARMC7*	0/119 (0.00%)	0/1337 (0.00%)	NA	NA	NA	NA
Wang et al. (2019)	*END1*	0/119 (0.00%)	0/1337 (0.00%)	NA	NA	NA	NA
Wang et al. (2019)	*HRAS*	0/119 (0.00%)	0/1337 (0.00%)	NA	NA	NA	NA
Wang et al. (2019)	*IRF2BPL*	0/119 (0.00%)	0/1337 (0.00%)	NA	NA	NA	NA
Wang et al. (2019)	*KN1A*	0/119 (0.00%)	0/1337 (0.00%)	NA	NA	NA	NA
Wang et al. (2019)	*KRAS*	0/119 (0.00%)	0/1337 (0.00%)	NA	NA	NA	NA
Wang et al. (2019)	*KRTAP9.1*	0/119 (0.00%)	0/1337 (0.00%)	NA	NA	NA	NA
Dai et al. (2016)	*PRSS42P*	0/119 (0.00%)	0/1337 (0.00%)	NA	NA	NA	NA
Wang et al. (2019)	*RAC1*	0/119 (0.00%)	0/1337 (0.00%)	NA	NA	NA	NA
Wang et al. (2019)	*RBMX*	0/119 (0.00%)	3/1337 (0.22%)	NA	NA	NA	NA
Wang et al. (2019)	*U2AF1*	0/119 (0.00%)	0/1337 (0.00%)	NA	NA	NA	NA
